# Comparative Performance of Hybrid and Elite Inbred Rice Varieties with respect to Their Source-Sink Relationship

**DOI:** 10.1155/2015/326802

**Published:** 2015-01-29

**Authors:** Md Moinul Haque, Habibur Rahman Pramanik, Jiban Krishna Biswas, K. M. Iftekharuddaula, Mirza Hasanuzzaman

**Affiliations:** ^1^Department of Agricultural Botany, Sher-e-Bangla Agricultural University, Dhaka 1207, Bangladesh; ^2^Department of Crop Botany, Bangladesh Agricultural University, Mymenshing 2202, Bangladesh; ^3^Plant Physiology Division, Bangladesh Rice Research Institute, Gazipur 1701, Bangladesh; ^4^Plant Breeding Division, Bangladesh Rice Research Institute, Gazipur 1701, Bangladesh; ^5^Department of Agronomy, Faculty of Agriculture, Sher-e-Bangla Agricultural University, Dhaka 1207, Bangladesh

## Abstract

Hybrid rice varieties have higher yield potential over inbred varieties. This improvement is not always translated to the grain yield and its physiological causes are still unclear. In order to clarify it, two field experiments were conducted including two popular indica hybrids (BRRI hybrid dhan2 and Heera2) and one elite inbred (BRRI dhan45) rice varieties. Leaf area index, chlorophyll status, and photosynthetic rate of flag leaf, postheading crop growth rate, shoot reserve translocation, source-sink relation and yield, and its attributes of each variety were comprehensively analyzed. Both hybrid varieties outyielded the inbred. However, the hybrids and inbred varieties exhibited statistically identical yield in late planting. Both hybrids accumulated higher amount of biomass before heading and exhibited greater remobilization of assimilates to the grain in early plantings compared to the inbred variety. Filled grain (%) declined significantly at delayed planting in the hybrids compared to elite inbred due to increased temperature impaired-inefficient transport of assimilates. Flag leaf photosynthesis parameters were higher in the hybrid varieties than those of the inbred variety. Results suggest that greater remobilization of shoot reserves to the grain rendered higher yield of hybrid rice varieties.

## 1. Introduction

Rice (*Oryza sativa* L.) is the main food crop of more than one-half of the world's population and grows worldwide. Rice production has to be increased 1% per annum to deliver sufficient rice for an evergrowing population of rice-consuming countries [1]. According to the Food and Agriculture Organization (FAO), hybrid rice technology is the key approach for the increase of global rice production [2]. It has a 15–30% advantage in yield over modern inbred rice varieties [3–5] but does not frequently exhibit higher yield potential [3, 6–11]. Higher grain yield of hybrid rice is an intricate outcome of genotype and environment interaction.

Higher dry matter accumulation before heading, longer leaf area duration, higher LAI, and higher photosynthetic capability at the grain filling period are the essentials for achieving higher yield of rice [6, 12–15]. However, the findings of previous studies on hybrid rice suggest cross talk about the physiological causes of its higher yield. Many investigators reported that greater biomass accumulation before heading and higher shoot reserve translocation are the decisive factors of higher yield in hybrids [16–21]. On the other hand, Yan et al. [22] and Yang et al. [8] reported that hybrid rice had higher productivity after heading but translocation of assimilates was inefficient (source use efficiency). Separately, Lafarge and Bueno [1] stated that higher yield of hybrid was the result of better sink regulation.

Poor grain filling is the cause of poor grain yield in hybrids [8, 10, 23]. Chlorophyll content reflects the intensity of plant photosynthetic capacity and the extent of leaf senescence [24]. Slow senescence and stronger photosynthetic capability of flag leaf, higher LAI at grain filling period, and higher postheading-CGR are the prerequisites for higher yield in hybrid rice [25–28]. The postanthesis decline in leaf area at specially the ripening stage was higher in the hybrid compared to the inbred [17]. Whereas, some investigators mentioned that the hybrid has the lower single-leaf photosynthetic rate throughout the whole growth period and greater LAI contributes to the heterosis in biomass production and grain yield [3, 29, 30]. So, the available information on photosynthetic capability of flag leaf, the roles of shoot reserve translocation, and postheading crop growth rate to higher grain yield of hybrid rice varieties are still controversial. Therefore, it is imperative to clarify this controversy on hybrid rice varieties.

Keeping the above facts in mind, the present investigation was undertaken to study source-sink relation, shoot reserve translocation, source activity, and yield attributes of hybrid rice varieties at gradually rising temperature environments in order to clarify whether the greater accumulated biomass induced-higher remobilization or postheading photosynthetic capability plays a key role in the higher yield of hybrids. In addition to this, the physiological characteristics of flag leaf in hybrid and elite inbred rice were also studied.

## 2. Materials and Methods

### 2.1. Experimental Sites, Design, and Crop Husbandry

The field experiments were conducted with two hybrids (BRRI hybrid dhan2 and Heera2) and one elite inbred (BRRI dhan45) rice varieties at the Experimental Farm of Sher-e-Bangla Agricultural University, Dhaka (23°77′N, 90°37′E and altitude of 9 m), and Bangladesh Rice Research Institute, Gazipur (23°99′N, 90°39′E and altitude of 8.4 m), Bangladesh in dry (*Boro*) seasons (December to May) of 2008-2009 and 2009-2010, respectively. Four planting dates were maintained, namely, 20 December, 5 January, 20 January, and 5 February. Soil of the experimental plots was medium highland with upper-middle range fertility. Properties of the top soil (0–15 cm.) of the experimental plots are summarized in Table 1. The experiments were laid out in a split-plot design with three replications, placing planting dates in the main-plots and varieties in the subplots. The unit plot size was 20 m^2^ (5.0 m × 4.0 m). One seedling (30 days old) was transplanted per hill maintaining 25 cm × 15 cm spacing. A single plot was comprised of 20 lines and each line contained 26 hills. A buffer (levee) of 0.5 m, 0.5 m, and 1.0 m was kept in between subplots, main-plots, and blocks, respectively. Cow-dung was applied @ 10 t ha^−1^ and chemical fertilizers as urea, triple superphosphate, muriate of potash, gypsum, and zinc sulphate were applied @ 270-130-120-70-10 kg ha^−1^, respectively [31]. Cow-dung was applied 15 days before land preparation. All the fertilizers were applied as basal except urea which was applied as top dressing in 3 equal installments at 15 days after transplanting (DAT), tillering stage (30 DAT), and panicle initiation stage (45 DAT). Climate of both experimental sites is similar. Temperature, solar radiation, and sunshine hours gradually increase from January to April. Rainfall is scanty or absent in early dry season (December-January). Weather conditions of the experimental seasons are shown in Table 2.

### 2.2. Plant Materials

The BRRI hybrid dhan2 is a popular indica hybrid rice variety developed by Bangladesh Rice Research Institute (BRRI) in 2008. It is recommended for cultivation in both dry seasons of Bangladesh. The Heera2 (HS-273) is the most popular indica hybrid rice variety, imported from China and occupied about 70% of total hybrid rice cultivated area in Bangladesh. Both hybrids mature in 140 to 145 days during the dry seasons (December to May) [31]. Also, BRRI dhan45 is a high yielding inbred variety for dry season and was developed by BRRI in 2005 through crossing between BRRI dhan2 and TETEP. Originally, this variety was developed from the breeding line BR5778-21-2-3 and its duration of growth ranges from 140 to 145 days.

### 2.3. Sampling and Measurements

Five plants were sampled at 10-day intervals starting from 20 DAT up to maturity. At first sampling, hills were selected from the third rows and the next two rows or three hills were left for subsequent sampling to minimize the border effect. Leaf area (LA) was measured by an automatic leaf area meter (Model: LI-3100, Li-COR, Lincoln, NE, USA.) just after removal of leaves to avoid rolling and shrinkage and transformed into leaf area index (LAI) according to Yoshida [32].

Ten plants were sampled from each plot at heading and at maturity. All the plant samples were separated into root, leaf blades (leaf), culm and sheath (stem), and panicles. Dry matter of each component was determined after drying at 72°C for 3 days to have constant weight. The preanthesis shoot (stem + leaf) reserve translocation was calculated by net loss in dry weight of vegetative organs between preanthesis and maturity [33].

Flag leaves were sampled from main tillers at 2, 9, 16, and 23 days after flowering and a 20 mg segment from middle portion of the flag leaf was used for chlorophyll analysis. Flag leaf chlorophylls were extracted using 80% acetone solution and were estimated with a double beam spectrophotometer (Model: U-2001, Hitachi, Japan) according to Witham et al. [34]. Flag leaves from the main tiller of five representative plants of each plot were used to measure photosynthetic rate with a portable photosynthesis analyzer (Model: LI-6400, Li-COR Inc. USA) at 2, 9, 16, and 23 days after flowering under clear sunshine from 11.00 am to 12.30 pm when photosynthetically active radiation at the top of the canopy ranged from 1300 to 1400 *μ*mol m^−2^ s^−1^. Then, the five readings were averaged for the plot.

Plants of 12 hills from each plot were harvested at maturity to record the yield contributing components. Plants from the central 6 m^2^ undisturbed area in each plot were harvested at maturity and grain yields were recorded.

Postheading crop growth rate as an index of plant productivity in grain filling period was calculated as the increase of plant dry matter per unit time and measured by taking the total dry matter at heading and at maturity [35]. Ratio of spikelets number to leaf area (at heading) and ratio of yield sink to leaf area (at heading) were calculated according to Zhao et al. [36]:

(1) Ratio  of  spikelets  no.  to  leaf  area  cm−2 =Spikelets  numberLeaf  area  at  heading,Ratio  of  yield  sink  to  leaf  area  mg cm−2 =Yield  sinkLeaf  area  at  heading.

Ratio of accumulated grain dry matter from current photosynthate (GDMCPn) to average leaf area (heading to maturity) was estimated using the following formula:

(2) Ratio  of  GDMCPn  to  LA  mg cm−2 =Yield  sink−panicle  wt.  at  heading+remobilizationLeaf  area  heading  to  maturity.

Current photosynthate accumulation panicle^−1^ (CPA) was computed using the following formula:

(3) CPA  panicle−1g =Av.  panicle  wt.  at  maturity  −+av.  remobilization  panicle−1av.  panicle  wt.  at  heading      +av.  remobilization  panicle−1.

### 2.4. Statistical Analysis

Collected data were put to analysis of variance (ANOVA) technique and the means were compared by the Duncan multiple range test (DMRT), using the statistical computer package program MSTAT-C [37]. Correlation and regression analyses were done using Microsoft Excel software.

## 3. Results

### 3.1. Grain Yield and Its Attributes

The grain yield and its attributes of the tested varieties at different planting dates have been shown in Table 3. In both the years, the maximum grain yield for all varieties was achieved at early planting dates (20 December) and there was a gradual decreasing trend observed in delayed planting. An almost similar trend was found in the case of panicle numbers m^−2^. Grain yield ranged from 4.44 to 7.42 t ha^−1^ for BRRI hybrid2, 4.37 to 8.03 t ha^−1^ for Heera2, and 4.57 to 6.16 t ha^−1^ for inbred BRRI dhan45 for all planting dates in both the years. The hybrids Heera2 produced significantly higher grain yield over inbred BRRI dhan45 at all planting dates except 5 February planting and an almost similar trend was observed for BRRI hybrid dhan2. The hybrids exhibited significantly lower number of panicles m^−2^ and on average 33.95% higher number of spikelets panicle^−1^ compared to the tested inbred, irrespective of planting date. Higher yield of Heera2 and BRRI hybrid dhan2 was attributed to the greater number of spikelets panicle^−1^ along with larger and heavier grain size. The studied hybrid and inbred varieties produced statistically similar grain yield at delayed planting (5 February) in 2008-09 and 2009-10, which was associated with rapid reduction of the number of filled grain percentage in hybrid varieties. Filled grain percentage of inbred BRRI dhan45 was more or less stable at different planting dates. This may be due to intrinsic genotypic characters or the well adaptability of the inbred BRRI dhan45 to the environment.

### 3.2. Flag Leaf Chlorophyll Content and Its Chlorophyll a : b Ratio

The studied hybrid varieties synthesized significantly higher amounts of chlorophyll and maintained higher chlorophyll a : b ratio in their flag leaf over inbred BRRI dhan45 (Table 4). Flag leaf chlorophyll content and chlorophyll a : b ratio gradually decreased in the hybrid and inbred varieties with advanced maturity. Reduction of chlorophyll content at 23 days after flowering compared to 2 days after flowering was 33 and 36% in hybrids and inbred, respectively. Planting dates had little influence on flag leaf chlorophyll content. Chlorophyll a : b ratio of the flag leaf was higher in both the hybrids. However, environmental influence on total chlorophyll content of flag leaf was relatively small.

### 3.3. Flag Leaf Photosynthetic Rate

Photosynthetic rate of flag leaf at 2, 9, 16, and 23 days after flowering is presented in Table 5. There was no significant difference among the tested hybrid and inbred varieties with respect to flag leaf photosynthetic rates at different days after flowering except at 23 days after flowering. Photosynthetic rate of flag leaf ranged from 33.29 to 34.75 *μ*mol CO_2 _m^−2^ s^−1^ among the varieties and from 32.25 to 35.25 *μ*mol CO_2 _m^−2^ s^−1^ among different planting dates at 2 days after flowering. The photosynthetic rate gradually decreased with the advance of grain filling period. At 23 days after flowering, Heera2 and BRRI hybrid dhan2 showed significantly higher photosynthetic rate than BRRI dhan45, but the studied hybrid and inbred varieties exhibited decreasing trend of photosynthetic rate of the flag leaf at 9, 16, and 23 days after flowering in all planting dates. The Heera2 and BRRI hybrid dhan2 showed considerable decrease in flag leaf photosynthetic rate 9 days after flowering in the 5 February planting. This shows the poor performance of Heera2 and BRRI hybrid dhan2 for the delayed planting dates.

### 3.4. Shoot Reserve Translocation

Dry matter accumulation at heading and its remobilization to the grain of the tested hybrid and inbred varieties at four planting dates for two year have been shown in Table 6. In each year, dry matter accumulation of hybrid Heera2 and BRRI hybrid dhan2 at the heading stage was higher than that of elite inbred BRRI dhan45, but the dry matter accumulation decreased 28% and 17.5% in the studied hybrids and inbred at 5 February planting compared to 20 December planting. The shoot reserve translocation (%) to grain varied among the different planting dates and gradually decreased with delayed planting. Studied hybrids exhibited 7.5% higher shoot reserve translocation to grain over inbred BRRI dhan45 at all planting dates except 5 February planting and the magnitude of decreasing assimilate remobilization rate to grain was steeper in hybrids and was in the order of Heera2 (60%) > BRRI hybrid dhan2 (41%) > inbred BRRI dhan45 (19%).

### 3.5. Leaf Area Index

**Figure 1:**
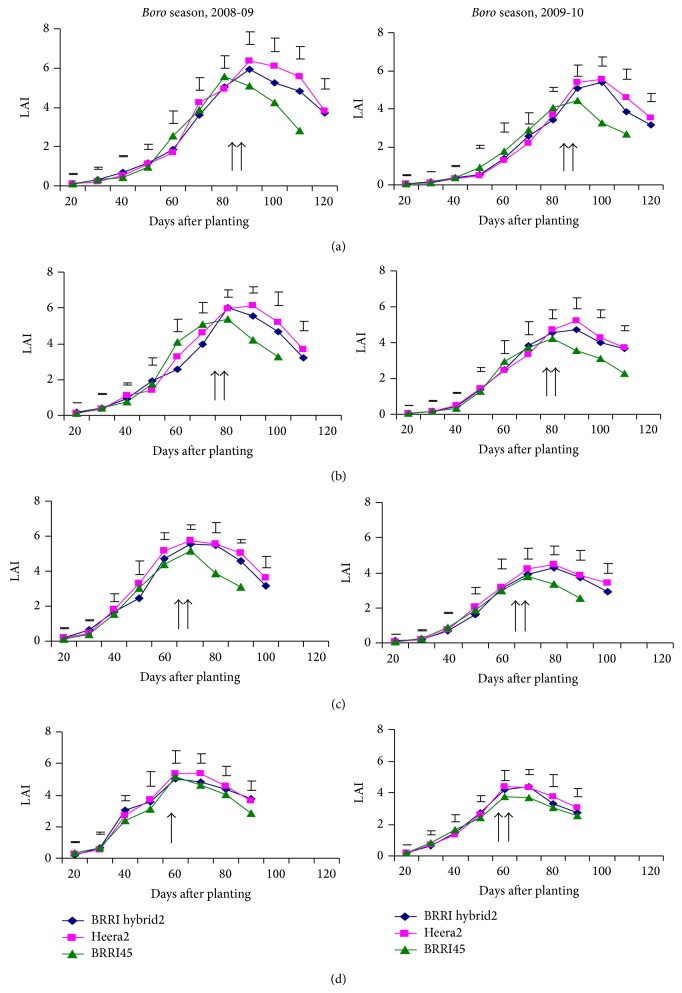
Leaf area index (LAI) of hybrid and inbred varieties at different DAT in dry seasons, 2008-09 and 2009-10, respectively. (a) 20 December planting, (b) 05 January planting, (c) 20 January planting, and (d) 05 February planting. Vertical bars represent standard error (*n* = 3). Arrows indicate start of heading stage.

Leaf area index (LAI) increased gradually in all the tested varieties in all the planting dates up to heading and in most of the cases the differences are nonsignificant. Thereafter, the reduction of LAI is greater in inbred than that of hybrids (Figure 1). As an outcome, the hybrid varieties sustained higher LAI after heading to maturity over inbred BRRI dhan45 regardless of planting dates. Days to heading decreased gradually with delayed planting and the magnitude of reduction was almost similar in the hybrid and inbred varieties. However, the maximum LAI was recorded from Heera2 (6.36) at heading stage followed by BRRI hybrid dhan2 (5.94) while it was significantly lower in BRRI dhan45 (5.10) at 20 December planting of 2008-09. The maximum value of LAI gradually decreased in hybrid and inbred varieties with delayed transplanting due to reduction of vegetative phase. The leaf area development of studied varieties at different planting dates in 2009-10 was more or less similar to the previous year. This result revealed that hybrid rice varieties maintained significantly greater LAI from heading to maturity stage compared to the inbred.

### 3.6. Source-Sink Relation and Postheading Crop Growth Rate

Ratio of spikelets number to leaf area (at heading), yield sink to leaf area (at heading), and grain dry matter accumulated from current photosynthetic assimilation to leaf area (average from heading to maturity) were reflected the source-sink relation in the studied hybrid and inbred varieties (Table 6). The Heera2 and BRRI hybrid dhan2 varieties had considerably larger sink and produced higher yield sink per unit leaf area over BRRI dhan45. Inbred BRRI dhan45 exhibited higher ratio of grain dry matter accumulated from current photosynthetic assimilation to leaf area (average from heading to maturity) compared to both hybrids. In delayed planting (5 February) yield sink per unit leaf area was significantly decreased in tested varieties. Both the studied varieties and the different planting dates showed almost similar trends in postheading crop growth rate as that of yield sink per unit leaf area. Interaction effect of variety and planting date were nonsignificant in all the cases. These results indicated that BRRI dhan45 has the genotypic superiority over the tested hybrids with respect to postheading photosynthetic assimilation per unit leaf area.

## 4. Discussion

Flag leaf is the main and most active photosynthetic source of grain yield in rice [32, 38, 39]. Chlorophyll content indicates photosynthetic efficiency of leaves [24, 40]. Tang et al. [41] reported that hybrid rice contains higher amounts of chlorophyll in their leaves. Islam et al. [42] found no variation in leaf chlorophyll content between hybrid and modern inbred varieties at* Boro* season (December to May) but in* Aman* season (July to December) recorded higher leaf chlorophyll content in hybrids over inbred rice varieties. In the present investigation flag leaf of the tested hybrid varieties contained higher amounts of chlorophyll and maintained higher chlorophyll a : b ratio over elite inbred BRRI dhan45. Fluctuation in flag leaf chlorophyll content was small among the different planting dates. It indicated that higher flag leaf chlorophyll content was the inherent characters of the studied hybrids. In this study there was no significant difference in flag leaf photosynthetic rate between the studied hybrid and inbred rice varieties, irrespective of planting dates. Cheng et al. [25], Poshtmasari et al. [27], and Tang et al. [41] reported higher photosynthetic capability of flag leaf in hybrid rice. However, Sinclair and Horie [29] and Peng et al. [3] reported that hybrid had lower single-leaf photosynthetic rate at grain filling phase as compared to inbred. Studied hybrid rice varieties showed slightly higher photosynthetic rate in flag leaf at 23 days after flowering. This may be due to the slow senescence of flag leaves and plants of the hybrids. Higher chlorophyll content in the flag leaves and its slow reduction toward maturity delayed senescence of the hybrid rice varieties (Table 4). This observation was partially consistent with Peng et al. [3]. Therefore, it seems consistent that higher flag leaf chlorophyll content and chlorophyll a : b ratio of hybrids play a significant role to increase photosynthetic rate during grain filling period.

**Figure 2:**
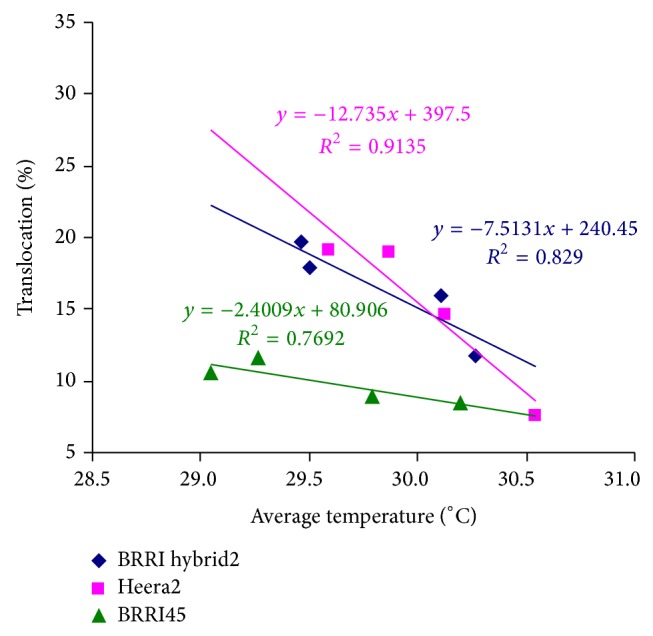
Relationship between the shoot reserve translocation percentage and average daily temperature from flowering to maturity. Average data of two dry seasons, 2008-2009 and 2009-2010 used.* R* ^2^ calculated as 5% level of significance.

In early plantings (20 December and 5 January), studied rice varieties took longer in the vegetative phase. Due to vigorous vegetative growth at the middle growth stage, tested hybrid varieties accumulated significantly higher amounts of dry matter at heading over the inbred. Accumulated larger dry matter triggered better assimilate remobilization at the grain filling stage. Both the hybrids, Heera2 and BRRI hybrid dhan2, clearly superseded the elite inbred BRRI dhan45 in respect of assimilate remobilization from shoot reserve in early planting (Table 6). A similar result was reported by several other investigators [1, 17, 19, 20, 43, 44]. Dry matter accumulation was steeper in Heera2 and BRRI hybrid dhan2 at early planting due to the low temperature induced-longer vegetative duration and efficient source activity. That means dry matter accumulation in the studied hybrid varieties before heading was highly thermosensitivity. Photosynthetic efficiency of the same genotypes varied markedly during different growth stages [45, 46]. The present study suggested accumulation of more dry matter before heading and its higher translocation into the developing grain during filling stage resulting in higher yield of hybrids over the modern inbred. This result was at par with the findings of Laza et al. [18], Jeng et al. [19], and Yang et al. [20]. The shoot reserve translocation was negatively correlated with the temperature of grain filling period. Hybrid rice varieties exhibited higher degree of sensitivity to temperature rising in regard to shoot reserve remobilization to grain compared to the inbred. For rising average daily temperature 1°C from 29°C, shoot reserve remobilization to the grain decreased at the rate of* ca.* 10.2% and 2.4% in the tested hybrid and inbred rice varieties, respectively (Figure 2, Table 7).

**Figure 3:**
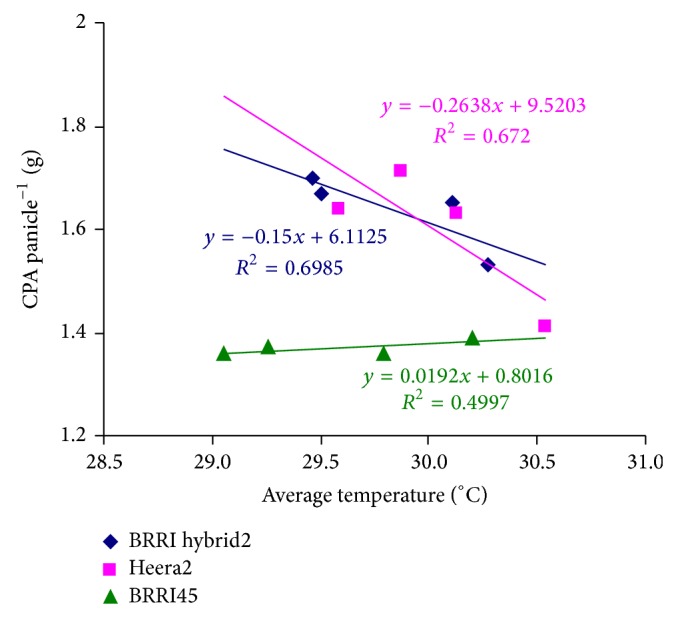
Relationship between current photosynthate accumulation (CPA) panicle^−1^ and average daily temperature from flowering to maturity. Average data of two dry seasons, 2008-2009 and 2009-2010 used.* R* ^2^ calculated as 5% level of significance.

In the case of early planting, higher biomass accumulation and rapid expansion of leaf area of the tested hybrids produced higher LAI just after heading and developed an efficient sink (large panicles). This higher leaf area index contributed to the supply of higher amount of photosynthate to the grain at the filling stage and finally contributed to the higher grain yield of hybrid Heera2 and BRRI hybrid dhan2. The ratio of grain dry matter accumulated from current photosynthate to average leaf area (from heading to maturity) was almost similar in hybrid and inbred rice varieties indicating that leaves other than the flag leaf of studied hybrid rice varieties had the lower photosynthetic capability at grain filling stage than inbred BRRI dhan45. This situation was aggravated by the rising temperature in delayed plantings. Besides these, shorter vegetative duration reduced dry matter accumulation and rising temperature impaired shoot reserves translocation. As a consequence, yield of hybrid varieties decreased rapidly compared to the inbred under delayed planting. Both the hybrid varieties showed relatively higher postheading-CGR at early planting due to their higher LAI at the grain filling period. Postheading photosynthetic dry matter accumulation panicle^−1^ in both the hybrids exhibited higher degree of thermosensitivity. Photosynthate accumulation panicle^−1^ decreased at the rate of* ca.* 0.2 g in the hybrids for rising average daily temperature 1°C from 29°C while this reduction rate was negligible in the inbred rice variety (Figure 3). It might be due to the intrinsic genetic trait or well adaptability of inbred BRRI dhan5 to environmental conditions. These results suggested that modern inbred BRRI dhan45 was efficient in source utilization at the grain filling stage under higher temperature. Grain filling is a deposition of starch from two sources: current photosynthate (60–100%) and remobilization (rest) from the reserve pool [23, 32, 47]. Grain filling index reflects the source-sink relationship [36]. Efficient assimilate supply to the grain from the source and the capacity of the sink to receive it determines the higher yield (grain filling percentage) and these processes depend highly upon environmental conditions [48, 49]. The percent of fertile spikelets gradually declines when daily mean temperature exceed 29°C [50] or 29.6°C [51]. Chakrabarti et al. [52] reported that pollen sterility gradually increased above 33°C. In spite of prevailing normal temperature one week before and after of flowering (data not shown) and normal appearance of spikelets, the number of filled grain as a percentage declined rapidly in studied hybrid varieties than in inbred BRRI dhan45 at delayed planting. This poor filled grain percentage was associated with lower postheading crop growth rate and also related to failure of assimilates supply to the spikelets due to rising temperature. Kobata et al. [48] reported that higher temperature limits the assimilate supply to meet the demand of grains. The photosynthetic rate of flag leaf significantly increased at flowering and then decreased rapidly in delayed planting (Table 4). It reveals that translocation of dry matter from shoot to grain was relatively inefficient for hybrids in delayed planting. This result confirms the findings of Yan et al. [22]. Yang et al. [53]; Yang et al. [8]; and Wu et al. [28] reported that the more dry matter in the vegetative organ at heading contributes little to the grain due to poor transportation and remobilization of stored assimilates and these account for poor grain filling rather than source limitation in super- and intersubspecific hybrids. Large sink size or sink strength of hybrids creating higher demand for photosynthetic assimilates increased photosynthetic rate in flag leaf initially at delayed planting. Individual grain weight remained unchanged in hybrids at delayed planting indicating that physiological activity of the sink was not affected significantly due to rising temperature. However, the studied hybrids were more vulnerable to rising temperature in respect to assimilate translocation to grain than that of the inbred variety. To avoid the adverse effect of higher temperature, the hybrid varieties should be transplanted at the onset of the dry season.

## 5. Conclusion

It is concluded that the tested hybrid rice varieties accumulate higher amount of dry matter before heading and maintain large LAI at the grain filling period compared to the inbred variety, irrespective of growing temperature. However, relatively high temperature impaired slow rate of remobilization and transportation of assimilates ultimately causes rapid reduction of the percentage of filled grain in the hybrids compared to the inbred variety.

## Figures and Tables

**Table 1:** Physical and chemical properties of the initial soil of the experimental plots.

Characteristics	Site 1^a∗^	Site 2^b∗^
	Value/type	Value/type
% Sand	29.97	13.08
% Silt	54.86	44.25
% clay	15.28	42.47
Textural class	Silty loam	Silty clay
P^H^	5.87	6.70
Organic matter (%)	1.43	1.20
Total N (%)	0.08	0.13
P (*μ*g/g soil)	5.58	7.49
S (*μ*g/g soil)	7.89	7.25
B (*μ*g/g soil)	0.16	0.12
Cu (*μ*g/g soil)	0.15	0.27
Fe (*μ*g/g soil)	2.80	4.35
Mn (*μ*g/g soil)	0.74	1.63
Zn (*μ*g/g soil)	0.43	0.34
Exchangeable K (meq/100 g soil)	0.10	0.17
Ca (meq/100 g soil)	1.43	2.40
Mg (meq/100 g soil)	0.35	0.87

^a^Department of Soil Science, Sher-e-Bangla Agricultural University, Dhaka 1207.

^b^Soil Science Division, Bangladesh Rice Research Institute (BRRI), Gazipur 1703.

^*^Red brown terrace soil type.

**Table 2:** Temperature, relative humidity (RH), total rainfall, solar radiation, and total sunshine hours of the experimental sites during dry seasons (December–May) in 2008-2009 and 2009-2010, respectively.

Months/years	Air temperature (°C)^c^			RH at 2 pm. (%)	Total rainfall (mm)	Solar radiation (Cal cm^2^)	Total sunshine (h)
	Min.	Max.	Av.				
^ a^1st season (2008-09)							
Dec 2008	16.90	25.57	21.24	70.5	0	257.0	121
Jan 2009	14.84	25.92	20.38	67.7	0	237.3	148
Feb 2009	17.31	29.79	23.55	63.0	1	347.9	178
Mar 2009	21.52	33.28	27.45	58.1	43	396.4	184
Apr 2009	25.86	35.58	30.72	68.8	13	410.1	255
May 2009	25.19	34.58	29.89	72.2	163	398.4	244
^ b^2nd season (2009-10)							
Dec 2009	14.40	25.29	19.85	74.9	0	213.0	148
Jan 2010	11.81	24.28	18.04	69.8	0	235.0	164
Feb 2010	14.49	29.05	21.81	71.8	15	339.9	194
Mar 2010	21.89	34.71	28.30	75.8	17	405.3	236
Apr 2010	25.02	35.29	30.16	70.3	58	394.4	215
May 2010	24.53	33.87	29.21	72.6	207	394.1	232

^a^Sher-e-Bangla Agricultural University, (Bangladesh Meteorological Department, Climate and Weather Division) Sher-e-Bangla Nagar, Dhaka 1207.

^b^Plant Physiology Division, Bangladesh Rice Research Institute, (BRRI) Gazipur 1703.

^c^Monthly average of daily temperatures.

**Table 3:** Yield and its attributes of hybrid and inbred rice varieties at different dates of planting in dryseasons, 2008-2009^¶^ and 2009-2010^¶¶^.

Variety × planting date	Panicles m^−2^		Spikelets panicle^−1^		Filled spikelets (%)		1000-grain weight (g)		Grain yield^a^ (t ha^−1^)	
	2008-09	2009-10	2008-09	2009-10	2008-09	2009-10	2008-09	2009-10	2008-09	2009-10
BRRI hybrid dhan2										
20 December	278.5	270.4	138.0	131.9	79.04ab	79.5ab	26.53	26.84	7.42ab	6.78ab
05 January	265.3	247.2	132.7	133.3	78.73ab	75.5bcd	26.93	26.76	6.84bc	6.10bc
20 January	246.5	235.1	133.6	130.1	76.23b	70.2ef	27.06	26.43	6.06cd	5.66cd
05 February	239.4	216.7	131.3	131.0	66.49c	66.4fg	26.96	26.88	5.34de	4.44f
Hybrid Heera2										
20 December	310.5	284.8	130.2	131.8	79.00ab	78.3abc	26.61	26.93	8.03a	7.31a
05 January	285.0	256.9	139.7	136.4	77.70ab	75.2cd	27.39	26.04	7.65ab	6.21bc
20 January	290.4	248.6	125.4	133.5	78.67ab	72.6de	27.03	26.54	7.28ab	5.59cd
05 February	260.7	241.3	128.8	122.3	64.81c	63.2g	27.21	27.14	5.12e	4.37f
Inbred BRRI45										
20 December	322.3	308.9	101.1	95.2	81.40ab	81.7a	25.86	25.54	6.16cd	5.52cd
05 January	303.8	278.7	105.2	97.08	82.39a	80.7a	25.54	25.16	5.74de	5.23de
20 January	292.9	276.7	99.3	98.51	81.28ab	78.8abc	26.05	25.22	5.62de	4.77ef
05 February	283.5	269.9	96.8	94.5	80.99ab	78.2abc	25.33	25.36	5.50de	4.57ef

Variety	∗∗	∗∗	∗∗	∗∗	∗∗	**∗∗**	∗∗	∗∗	∗∗	∗∗
Planting date	∗	∗∗	NS	NS	NS	**∗∗**	NS	NS	∗∗	∗∗
CV (%)	8.71	9.30	6.64	8.80	4.13	3.31	3.86	3.06	8.24	7.42

Within a column for each site, means followed by the same letters are not significantly different according to DMRT at 5% level of significance. ^¶^site SAU, ^¶¶^Dhaka. site BRRI, Gazipur.

NS: nonsignificant at 5% level of significance.

^*^Significant at 5% level of significance.

^**^Significant at 1% level of significance.

^a^14% moisture contained in yield.

**Table 4:** Comparison of flag leaf chlorophyll content and chlorophyll a : b ratio among hybrid and inbred rice varieties at different dates of planting in dryseason, 2009-2010^¶^.

Variety/planting date	Total chlorophyll (mg g^−1^)				Chlorophyll a : b			
	2DAF	9DAF	16DAF	23DAF	2DAF	9DAF	16DAF	23DAF
Hybrids								
BRRI hybrid dhan2	2.65b	2.53a	2.16b	1.77b	3.18a	2.97a	2.41b	2.09
Heera2	2.80a	2.57a	2.29a	1.87a	3.22a	2.88a	2.51a	2.13
Inbred BRRI dhan45	2.05c	1.80b	1.49c	1.30c	2.88b	2.63b	2.39b	1.99
Planting dates								
20 December	2.42	2.32	2.05	1.71a	3.15	2.90	2.48	2.14
05 January	2.52	2.28	2.02	1.65ab	3.16	2.85	2.51	2.05
20 January	2.52	2.33	1.97	1.67a	3.07	2.77	2.39	2.06
05 February	2.53	2.28	1.89	1.56b	2.97	2.77	2.37	2.05
Interaction								
Variety × planting date	NS	NS	NS	NS	NS	NS	NS	NS
CV (%)	6.21	5.73	7.30	6.69	5.01	5.79	3.93	6.65

Within a column, means followed by the same letter(s) are not significantly different at 5% level of probability by DMRT. ^¶^site BRRI, Gazipur.

NS: nonsignificant at 5% level of significance.

DAF: days after flowering.

**Table 5:** Comparison of flag leaf photosynthetic rate among hybrid and inbred rice varieties at different dates of planting in dryseason, 2009-2010^¶^.

Variety/planting date	Photosynthetic rate (*μ*mol CO_2_ m^−2^ s^−1^)			
	2DAF	9DAF	16DAF	23DAF
Variety				
BRRI hybrid dhan2	34.14	28.83	21.59	15.01a
Heera2	34.75	29.22	21.28	15.23a
Inbred BRRI dhan45	33.29	28.97	20.55	13.02b
Planting date				
20 December	32.25b	30.40a	22.26	14.75
05 January	34.12ab	30.11a	21.68	15.19
20 January	34.60a	29.25a	20.99	14.08
05 February	35.25a	26.27b	19.63	13.63
Interaction				
Variety × planting date	NS	NS	NS	NS
CV (%)	4.64	5.50	9.73	6.14

Within a column, means followed by the same letter(s) are not significantly different at 5% level of probability by DMRT. ^¶^site BRRI, Gazipur.

NS: nonsignificant at 5% level of significance.

DAF: days after flowering.

**Table 6:** Preanthesis dry matter accumulation in shoot and its translocation to the grain of hybrid and inbred rice varieties at different dates of planting in dryseasons, 2008-2009^¶^ and 2009-2010^¶¶^.

Variety × planting dates	Shoot dry matter at preanthesis (g m^−2^)		Shoot dry matter at maturity (g m^−2^)		Changes in shoot dry matter (g m^−2^)		Shoot reserve translocation (%)	
	2008-09	2009-10	2008-09	2009-10	2008-09	2009-10	2008-09	2009-10
BRRI hy. dhan2								
20 December	846.95	729.92	691.99	575.55	154.95ab	155.37a	18.23a	21.10a
05 January	807.36	713.06	662.39	587.56	145.18ab	125.50ab	17.96ab	17.76bc
20 January	649.59	661.62	544.86	557.51	104.73cd	104.10b	16.14b	15.78c
05 February	584.34	559.51	510.55	498.48	73.79de	61.03cd	12.51c	10.84d
Hybrid Heera2								
20 December	925.37	784.71	746.50	637.06	178.87a	147.65a	19.39a	18.68ab
05 January	827.57	731.06	666.04	605.29	158.53ab	133.87ab	19.53a	18.23bc
20 January	741.76	662.03	603.25	592.38	138.51bc	69.65c	18.68ab	10.57d
05 February	615.54	602.53	595.45	560.61	53.43e	41.92cd	8.12d	7.00e
Inbred BRRI45								
20 December	708.36	596.02	622.57	534.09	85.76de	61.93cd	10.85cd	10.29d
05 January	729.69	569.71	640.89	507.80	61.81e	61.8cd	12.11c	11.03d
20 January	610.48	549.82	540.34	513.47	70.14de	36.34d	11.32c	6.54e
05 February	557.94	518.73	502.82	481.91	55.12e	36.82d	10.01cd	7.03e

Variety	∗∗	∗∗	∗	∗∗	∗∗	∗∗	∗∗	∗∗
Planting date	∗∗	∗	∗∗	NS	∗∗	∗∗	∗∗	∗∗
CV (%)	8.82	8.06	8.97	6.59	19.52	21.11	12.69	11.50

Within a column, means followed by the same letter(s) are not significantly different at 5% level of probability by DMRT. ^¶^site SAU, Dhaka. ^¶¶^site BRRI, Gazipur.

NS: nonsignificant at 5% level of significance.

^*^Significant at 5% level of significance.

^**^Significant at 5% level of significance.

**Table 7:** Source-sink relation and postheading crop growth rate of hybrid and inbred rice varieties at different dates of planting in dryseasons, 2008-2009^¶^ and 2009-2010^¶¶^.

Variety/planting dates	Ratio of spikelets number to LA^a^ (cm^−2^)		Ratio of yield sink to LA^a^ (mg cm^−2^)		Ratio of GDMCPn to LA^b^ (mg cm^−2^)		Postheading CGR (gm^−2^ d^−1^)	
	2008-09	2009-10	2008-09	2009-10	2008-09	2009-10	2008-09	2009-10
Variety								
BRRI hybrid dhan2	0.62a	0.68a	12.37ab	13.78a	10.03b	10.70b	15.05b	15.57
Heera2	0.65a	0.66a	13.05a	13.45a	10.30b	10.33b	16.21a	15.48
Inbred BRRI dhan45	0.56b	0.61b	12.03b	12.31b	11.56a	12.19a	14.42b	14.77
Planting date								
20 December	0.63a	0.69a	13.33a	14.18a	10.75	11.40	15.91ab	16.15a
05 January	0.63a	0.66ab	12.71a	13.28a	10.70	10.93	16.09a	15.76a
20 January	0.61ab	0.65ab	12.57a	13.45a	10.75	11.23	15.10b	15.27ab
05 February	0.57b	0.61b	10.87b	11.82b	10.33	10.69	13.90c	13.90b
Interaction								
Planting date × variety	NS	NS	NS	NS	NS	NS	NS	NS
CV (%)	9.90	8.83	8.31	7.97	9.36	7.09	8.27	10.26

Within a column, means followed by the same letter(s) are not significantly different at 5% level of probability by DMRT. ^¶^site SAU, Dhaka. ^¶¶^site BRRI, Gazipur.

^a^At heading.

^b^Average from heading to maturity.

NS: nonsignificant at 5% level of significance.

LA: leaf area.

GDMCPn: grain dry matter from current photosynthate.

## References

[1] Lafarge T., Bueno C. S. (2009). Higher crop performance of rice hybrids than of elite inbreds in the tropics: 2. Does sink regulation, rather than sink size, play a major role?. *Field Crops Research*.

[2] Virmami S. S., Bhuiyna S. I., Karim A. N. M. R. (1999). Shifting the yield frontier with hybrid rice. *Increasing Rice Production in Bangladesh—Challenges and Strategies*.

[3] Peng S., Cassman K. G., Virmani S. S., Sheehy J., Khush G. S. (1999). Yield potential trends of tropical rice since the release of IR8 and the challenge of increasing rice yield potential. *Crop Science*.

[4] Julfiquar A. W., Hasan M. J., Azad A. K., Nurunnab A. M. (2002). Research and development of hybrid rice in Bangladesh. *Hybrid Rice in Bangladesh: Progress and Future Strategies*.

[5] Julfiquar A. W. (2009). BRRI: research and development of hybrid rice. *The Guardian*.

[6] Horie T., Lubis I., Takai T., Mew T. W., Brar D. S., Peng S., Dawe D., Hardy B. (2003). Physiological traits associated with higher yield potential in rice. *Rice Science: Innovations and Impact for Livelihood: Proceedings of the International Rice Research Conference 16-19 September 2002*.

[7] Ying J., Peng S., He Q. (1998). Comparison of high-yield rice in tropical and subtropical environments I. Determinants of grain and dry matter yields. *Field Crops Research*.

[8] Yang J., Peng S., Zhang Z., Wang Z., Visperas R. M., Zhu Q. (2002). Grain and dry matter yields and partitioning of assimilates in japonica/indica hybrid rice. *Crop Science*.

[9] Nayak B. C., Dalei B. B., Choudhury B. K. (2003). Response of hybrid rice (*Oryza sativa*) to date of planting, spacing and seedling rate during wet season. *Indian Journal of Agronomy*.

[10] Ao H. J., Wang S. H., Zou Y. B. (2008). Study on yield stability and dry matter characteristics of super hybrid rice. *Scientia Agricultura Sinica*.

[11] Abou Khalifa A. A. (2009). Physiological evaluation of some hybrid rice varieties under different sowing dates. *Australian Journal of Crop Science*.

[12] Lin Q. H. Studies on ‘grain-leaf ratio’ of population and cultural approaches of high yield in rice plants. *Quality of Crop Population*.

[13] Dutta R. K., Basir-Mia M. A., Khanum S. (2002). Plant architecture and growth characteristics of fine grain and aromatic rice and their relation with grain yield. *International Rice Commission Newsletter*.

[14] Sheehy J. E., Mitchell P. L., Ferrer A. B. (2004). Bi-phasic growth patterns in rice. *Annals of Botany*.

[15] Katsura K., Maeda S., Horie T., Shiraiwa T. (2007). Analysis of yield attributes and crop physiological traits of Liangyoupeijiu, a hybrid rice recently bred in China. *Field Crops Research*.

[16] Yan Z. (1981). Studies on the production and distribution of dry matter in high-yielding populations of hybrid rice. *Acta Agronomica Sinica*.

[17] Khan M. N. A., Murayama S., Ishimine Y., Tsuzuki E., Motomura K., Nakamura I. (1998). Growth and yield in F_1_ hybrids of rice ( *Oryza sativa* L.). *Japanese Journal of Tropical Agriculture*.

[18] Laza M. R. C., Peng S., Akita S., Saka H. (2003). Contribution of biomass partitioning and translocation to grain yield under sub-optimum growing conditions in irrigated rice. *Plant Production Science*.

[19] Jeng T. L., Tseng T. H., Wang C. S., Chen C. L., Sung J. M. (2006). Yield and grain uniformity in contrasting rice genotypes suitable for different growth environments. *Field Crops Research*.

[20] Yang W., Peng S., Laza R. C., Visperas R. M., Dionisio-Sese M. L. (2007). Grain yield and yield attributes of new plant type and hybrid rice. *Crop Science*.

[21] Chen S., Zeng F., Pao Z., Zhang G. (2008). Characterization of high-yield performance as affected by genotype and environment. *Journal of Zhejiang University of Science B*.

[22] Yan J. M., Zhai H. Q., Zhang R. X., Jiao D. M., Chen B. S., Zhan H. S. (2001). Study on characteristics of photosynthesis and assimilates transportation in heavy ear hybrid rice (*Oryza sativa* L.). *Acta Agronomica Sinica*.

[23] Yang J., Zhang J. (2010). Grain-filling problem in ‘super’ rice. *Journal of Experimental Botany*.

[24] Wang R. F., Zhang Y. H., Qian L. S., Wu L. H., Tao Q. N. (1999). Studies on diagnostics of nitrogen in rice using chlorophyll meter. *Journal of Zhejiang Agricultural University*.

[25] Cheng S. H., Cao L. Y., Chen S. G. (2005). Conception of late-stage vigor super hybrid rice and its biological significance. *Chinese Journal of Rice Science*.

[26] Takai T., Matsuura S., Nishio T., Ohsumi A., Shiraiwa T., Horie T. (2006). Rice yield potential is closely related to crop growth rate during late reproductive period. *Field Crops Research*.

[27] Poshtmasari H. K., Pirdashti H., Nasiri M., Bahmanyar M. A. (2007). Study the effect of nitrogen fertilizer management on dry matter remobilization of three cultivars of rice (*Oryza sativa* L.). *Pakistan Journal of Biological Sciences*.

[28] Wu W.-G., Zhang H.-C., Qian Y.-F. (2008). Analysis on dry matter production characteristics of super hybrid rice. *Rice Science*.

[29] Sinclair T. R., Horie T. (1989). Leaf nitrogen, photosynthesis, and crop radiation use efficiency: a review. *Crop Science*.

[30] Laza M. R. C., Peng S., Sanico A. L., Visperas R. M., Akita S. (2001). Higher leaf area growth rate contributes to greater vegetative growth of F_1_ rice hybrids in the tropics. *Plant Production Science*.

[31] BRRI (2008). *Adunik Dhaner Chash (Modern Rice Cultivation)*.

[32] Yoshida S. (1981). Physiological analysis of rice yield. *Fundamentals of Rice Crop Science*.

[33] Bonnett G. D., Incoll L. D. (1992). The potential pre-anthesis and post-anthesis contributions of stem internodes to grain yield in crops of winter barley. *Annals of Botany*.

[34] Witham H., Blaydes D. F., Devlin R. M. (1986). *Exercises in Plant Physiology*.

[35] Choudhury D. A., Hamid H., Miah G. U., Haque M. M. (1998). Phenology, growth and yield ability of modern and old rice cultivars of different maturity groups. *Bangladesh Agronomy Journal*.

[36] Zhao B. H., Wang P., Zhang H. X., Zhu Q. S., Yang J. C. (2006). Source-sink and grain filling characteristics of two-line hybrid rice Yangliangyou 6. *Rice Science*.

[37] Russell D. F. (1986). *MSTAT-C Package Program*.

[38] Cao S. Q., Zhao Y. Q., Wen J. L., Wang S. A., Zhang R. X. (2000). Studies on photosynthesis in flag leaves and its relation to grain filling course of high yield wheat. *Scientia Agricultura Sinica*.

[39] Reo S. D. (1997). Flag leaf a selection criterion for exploiting potential yields in rice. *Indian Journal of Plant Physiology*.

[40] Hotta K. M., Satoh K., Katoh S. (1987). Relationship between photosynthesis and chlorophyll content during leaf senescence of rice seedlings. *Plant and Cell Physiology*.

[41] Tang W.-B., Zhang G.-L., Xiao Y.-H. (2010). Physiological and biochemical characteristics in flag leaves of the C Liangyou series of rice hybrid combinations at late growth stages. *Rice Science*.

[42] Islam M. S., Bhuiya M. S. U., Rahman S., Hussain M. M. (2010). Evaluation of SPAD and LCc based nitrogen management in rice (*Oryza sativa* L.). *Bangladesh Journal of Agricultural Research*.

[43] Qi C. H. (1993). Analysis on source-sink relationship and its regulation techniques of hybrid rice combinations. *Journal of Jiangxi Agricultural University*.

[44] Peng S., Yang J., Gracia F. V., Virmani S. S., Siddiq E. A., Muralidaran K. (1998). Physiology-based crop management for yield maximization of hybrid rice. *Advances in Hybrid Rice*.

[45] Sarker M. A. Z., Murayama S., Ishimine Y., Tsuzuki E. (2001). Heterosis in photosynthetic characters and dry matter production in F_1_ hybrids of rice. *Nippon Sakumotsu Gakkai Koenkai Yoshi, Shiryoshu*.

[46] Tang J. J., Chen H., Katsuyoshi S. (2002). Varietial differences in photosynthetic characters and chlorophyll fluorescence induction kinetics parameters among intergeneric progeny drive from *Oryza* x *Sorghum*, its parents and hybrid rice. *Journal of Zhejiang University of Science B*.

[47] Samonte S. O. P. B., Wilson L. T., McClung A. M., Tarpley L. (2001). Seasonal dynamics of nonstructural carbohydrate partitioning in 15 diverse rice genotypes. *Crop Science*.

[48] Kobata T., Sugawara M., Takatu S. (2000). Shading during the early grain filling period does not affect potential grain dry matter increase in rice. *Agronomy Journal*.

[49] Kobata T., Uemuki N. (2004). High temperatures during the grain-filling period do not reduce the potential grain dry matter increase of rice. *Agronomy Journal*.

[50] Oh-e I., Saitoh K., Kuroda T. Effects of rising temperature on growth, yield and dry-matter production of rice grown in the paddy field.

[51] Jagadish S. V. K., Craufurd P. Q., Wheeler T. R. (2007). High temperature stress and spikelet fertility in rice (*Oryza sativa* L.). *Journal of Experimental Botany*.

[52] Chakrabarti B., Aggarwal P. K., Singh S. D., Nagarajan S., Pathak H. (2010). Impact of high temperature on pollen germination and spikelet sterility in rice: comparison between basmati and non-basmati varieties. *Crop and Pasture Science*.

[53] Yang J. C., Zhu Q. S., Wang Z. Q., Liang Y. Z. (1997). Photosynthetic characteristics, dry matter accumulation and its translocation in inter-sub-specific hybrid rice. *Acta Agronomica Sinica*.

